# Supplementary material for: Phosphorus Supplementation Enhances Growth and Antioxidant Defense Against Cadmium Stress in Cotton
Source: Antioxidants (Basel). 2025 Jun 5;14(6):686. doi: 10.3390/antiox14060686 (PMC12189091; doi:10.3390/antiox14060686)
Supplement: Supplementary file 1 [file antioxidants-14-00686-s001.zip › antioxidants-3649438-supplementary.pdf]

Table S1. Principal component analysis (PCA) biplot of various studied traits of Jimian169 and DES926 under LP + Cd (0.01 mM KH_2PO_4 + 0.5 mM $CdCl_2$), LP + CK (0.01 mM KH_2PO_4 + 0 mM $CdCl_2$), NP + Cd (1 mM KH_2PO_4 + 0.5 mM $CdCl_2$), NP + CK (1 mM KH_2PO_4 + 0 mM $CdCl_2$). SL; shoot length, RDM; root dry matter, SDM; shoot dry matter, TDM; total dry matter, RL; root length, RSA; root surface area, RD; root diameter, RV; root volume, Pn; photosynthetic rate, gs; stomatal conductance, E; transpiration rate, Ci; intercellular CO_2 concentration, Chl a; chlorophyll a content, Chl b; chlorophyll b content, chlorophyll a + b, CAR; carotenoid contents, RP; root phosphorus concentrations, SP; shoot phosphorus concentration, RPA; root phosphorus accumulation, SPA; shoot phosphorus accumulation, PUpE; phosphorus uptake efficiency, PUtE; phosphorus utilization efficiency, RMDA; root malondialdehyde content, SMDA; shoot malondialdehyde content, RO_2; root superoxide anion, SO_2; shoot superoxide anion, RH_2O_2; root hydrogen peroxide, SH_2O_2; shoot hydrogen peroxide, RSOD; root superoxide dismutase, SSOD, shoot superoxide dismutase, RPOD; root peroxidase, SPOD; shoot peroxidase, RCAT; root catalase, SCAT; shoot catalase, RFAA; root free amino acids, SFAA; shoot free amino acids, RTSP; root total soluble proteins, STSP; shoot total soluble proteins, RTSS; root total soluble sugars, and STSS; shoot total soluble sugars.

**Traits**	**PC1**	**PC2**
SL	0.17	0.12
RD	0.17	0.08
SD	0.17	0.05
TD	0.17	0.06
RL	0.17	0.04
RSA	0.05	-0.37
RDM	0.14	-0.12
RV	0.06	-0.35
Pn	0.17	0.06
E	0.17	0.06
Gs	0.17	0.14
Ci	-0.17	-0.05
Chl A	0.16	0.11
Chl B	0.16	0.12
Chl A+B	0.16	0.12
Car	0.17	-0.13
RP	0.17	0.11
SP	0.17	0.12
RPA	0.17	0.09
SPA	0.17	0.06
PUpE	0.17	0.05
PUtE	0.17	0.03
RSOD	0.17	0.08
SSOD	0.17	-0.01
RPOD	0.15	0.22
SPOD	0.14	0.24

RCAT	0.17	0.03
SCAT	0.17	0.03
RMDA	-0.17	0.00
SMDA	-0.17	-0.09
RFAA	0.14	-0.25
SFAA	0.13	-0.29
RTSP	-0.15	0.18
STSP	-0.13	0.20
RTSS	-0.12	0.28
STSS	-0.13	0.23
RO_2	-0.16	0.05
SO_2	-0.16	0.05
RH_2O_2	-0.15	0.22
SH_2O_2	-0.15	0.22
Eigenvalue	32.25	5.53
Percentage of Variance	80.63%	13.81%
Cumulative	80.63%	94.45%
